# *CaSBP12* is implicated in pepper’s defense resistance to *Phytophthora capsici* infection associated with the SA signaling pathway

**DOI:** 10.1186/s12870-025-07858-z

**Published:** 2025-12-02

**Authors:** Huai-Xia Zhang, Shuang Wu, Yu-Ting Yang, Abid Khan, Chun-Hui Wu, Fei-Fei Pan, Bi-Hua Chen, Xin-Zheng Li, Bing Hu, Zhen-Hui Gong

**Affiliations:** 1 College of Horticulture and Landscape Architecture, Henan Institute of Science and Technology, Xinxiang, Henan P.R. China; 2 College of Horticulture, Northwest A&F University, No.3 TaichengRoad, Yangling, Shaanxi 712100 P. R. China; 3 School of Wine & Horticulture, Ningxia University, Yinchuan, 715100 China; 4 Department of Horticulture, The University of Haripur, Haripur, 22620 Pakistan; 5 Henan Provincial Agricultural and Rural Science and Technology Education Center, Zhengzhou, China

**Keywords:** Pepper, CaSBP12, Phytophthora capsici, Defense-related genes, SA signaling pathway

## Abstract

**Background:**

Pepper is a crucial vegetable crop with significant economic value, yet its yield and quality are severely affected by pepper *Phytophthora* Blight. Our previous research indicated that *CaSBP12* negatively regulates pepper resistance to *Phytophthora capsici* (*P. capsici*) infection. However, the precise role of *CaSBP12* in the defense response against *P. capsici* remains unclear. Thus, it is essential to investigate the defense mechanisms by which *CaSBP12* contributes to pepper resistance against *P. capsici* infection.

**Results:**

In this study, silencing *CaSBP12* significantly increased the expression of defense-related genes (*CaBPR1*, *CaSAR8.2*, *CaDEF1*, *CaPO1*) in *CaSBP12*-silenced plants compared to controls after *P. capsici* inoculation. Even under normal conditions, these genes exhibited higher expression levels in *CaSBP12*-silenced plants relative to controls. Conversely, these genes were downregulated in *CaSBP12*-overexpressing pepper plants under stress-free conditions. Therefore, we hypothesized that *CaSBP12* mediates the *P. capsici* defense response through these genes. Further research involved salicylic acid (SA) and jasmonic acid (JA) signaling pathway mutants (sid2, coi1-21, coi1-22) and the *NahG* gene (salicylate hydroxylase, inhibiting SA accumulation in plants). Without treatment, SA pathway genes *AtNPR1*, *AtTGA6*, *AtPR1*, and *AtSARD1* were significantly higher in *CaSBP12*-overexpressing *Arabidopsis thaliana* lines than in wild-type, while *AtNPR3*, *AtTGA5*, *AtPAD4*, *AtNPR4*, and *AtNDR1* were lower. In the sid2 mutant, *CaSBP12* promoted the expression of SA pathway genes except for *AtPR1*, which was suppressed. In coi1-21 and coi1-22 mutants, *CaSBP12* promoted *AtPR1* expression and suppressed the JA pathway gene *AtPDF1.2*. In *NahG* and *CaSBP12* co-expression lines, SA pathway genes were higher compared to *NahG*-overexpressing lines, while *AtPR1*, *AtNDR1*, *AtSARD1*, and *AtCBP60g* levels were lower.

**Conclusions:**

*CaSBP12* may participate in plant defense responses by regulating the expression of defense-related genes. It may inhibit upstream SA signaling genes *NDR1*, *PAD4*, and *EDS5* while promoting *NPR1* expression and inhibiting *NPR3* and *NPR4*, thereby regulating *PR* gene expression to participate in plant defense responses. These findings lay the foundation for further elucidating the molecular mechanisms of *CaSBP12* in pant defense response against *P. capsici* infection.

**Supplementary Information:**

The online version contains supplementary material available at 10.1186/s12870-025-07858-z.

## Introduction

Pepper (*Capsicum annuum* L.), a globally important vegetable crop with substantial economic value, currently occupies approximately 4.68 million hectares of cultivated land worldwide, yielding 82.82 million tons annually [1]. However, pepper cultivation faces substantial challenges from various pathogenic diseases, including fusarium wilt, viral infections, anthracnose, and *Phytophthora* blight. Notably, *Phytophthora* blight, caused by the oomycete pathogen *P. capsici*, poses a particularly severe threat [2]. This pathogen targets the roots, stems, leaves, flowers, and fruits of pepper plants, leading to plant mortality and substantial economic repercussions. Under favorable environmental conditions for the pathogen, *P. capsici* can cause complete yield failure in tropical and subtropical regions, resulting in annual global economic losses estimated at $1–10 billion [3, 4]. Current management strategies rely heavily on chemical fungicides, but these approaches risk the evolution of pathogen resistance and environmental contamination. Breeding disease resistant varieties is the most economical, safe, and effective measure to prevent and control pepper *Phytophthora* blight, and studying the regulatory mechanism of disease-related genes is crucial for such molecular improvement and breeding of pepper for *Phytophthora* blight resistance.

Salicylic acid (SA) plays a crucial role in plant defense responses against various pathogenic infections. In bacteria, SA biosynthesis can be catalyzed by isochorismate synthase and pyruvate lyase [5]. However, *Arabidopsis* contains only two isochorismate synthase genes and lacks pyruvate lyase homologs [5]. The *SA-DEFICIENT2* (*SID2*) gene encodes ISOCHORISMATE SYNTHASE1 (ICS1), a key enzyme in the SA biosynthesis pathway [6]. The *NPR1* gene, acting downstream of the SA-mediated disease resistance signaling pathway, serves as a critical regulator of SA-dependent signaling [7]. *NPR1* overexpression in tobacco and tomato enhances disease resistance in transgenic lines [8, 9]. *Arabidopsis* possesses five *NPR1* homologs. Notably, loss-of-function mutation of *NPR3* increases *PR-1* gene expression, while simultaneous loss-of-function mutation of *NPR3* and *NPR4* resulted in even higher *PR-1* expression. Furthermore, npr3-npr4 double mutant exhibits enhanced resistance to virulent bacterial and oomycete pathogens. Thus, *NPR3* and *NPR4* likely negatively regulate *PR* gene expression and plant pathogen resistance by interacting with *TGA2* [10]. These genes have been identified as SA receptors in plant immune signaling [11]. Additionally, *NPR1* is essential for induced systemic acquired resistance [11]. SA exerts dual regulatory effects: it suppresses *NPR3* and *NPR4* expression, thereby inhibiting their interaction with TGA transcription factors and relieving repression of defense-related genes; simultaneously, it promotes *NPR1* expression, which interacts with TGA factors to activate defense-related genes and enhance pathogen resistance [12]. As functionally redundant SA receptors, *NPR3* and *NPR4* act antagonistically to *NPR1* in early SA-mediated defense signaling [12]. NPR1 interacts with TGA2, TGA5, and TGA6 transcription factors [13]. While these *TGA* transcription factors exhibit functional redundancy, the tga2-tga5-tga6 triple mutant exhibited suppressed SA-induced *PR* gene expression and compromised disease resistance, unlike single mutants [13]. Notably, these *TGA* factors negatively regulate basal *PR-1* expression in uninduced conditions [13]. *MAPK4* negatively regulates SA signaling, with its mutant exhibiting constitutive systemic acquired resistance and elevated SA levels [14]. Mutations in *EDS1* or *PAD4* can suppress SA-mediated defense signaling [15]. Additionally *EDS5* loss-of-function mutation impairs SA biosynthesis, while *EDS12*, acting downstream of SA, is essential for systemic acquired resistance [15].

Pathogen infection upregulates *ICS1* expression, promoting SA biosynthesis [16]. The calmodulin-binding protein *CBP60g* and *SAR DEFICIENT1* (*SARD1*) transcriptionally regulate *ICS1* expression and SA biosynthesis [16]. *SARD1* knockout reduces basal and systemic acquired resistance, while its overexpression activates defense responses. The sard1-cbp60g double mutant shows impaired pathogen-induced *ICS1* expression and SA accumulation during both resistance phases [16]. These genes also negatively regulate NLR-mediated immunity and exhibit partial functional redundancy in SA biosynthesis, with *CBP60g* functioning primarily in early defense and *SARD1* in later stages [17, 18]. *TGA1* and *TGA4* regulate SA biosynthesis by controlling *SARD1* and *CBP60g* transcription, as indicated by the reduced expression of these genes and consequent SA reduction in tga1-tga4 mutants [18].

Additionally, jasmonic acid (JA) signaling plays vital roles in plant defense, development, and metabolism [19]. The JA-insensitive coi1 mutant was first identified in *Arabidopsis* in 1994 [20]. *COI1*, an F-box protein related to the auxin receptor *TIR1* [21], functions as part of the *SCF*^*COI1*^ *E3* ubiquitin ligase complex (comprising *ASK1*, *ASK2*, *CUL1*, and *RBX1*) to mediate JA signaling [22]. This complex promotes degradation of JA signaling repressors, thus activating JA-responsive transcription [23]. Transgenic plants expressing truncated *JAZ1* (lacking the C-terminal domain) exhibit COI1-like JA insensitivity [24]. JA treatment induces *COI1*-dependent proteasomal degradation of *JAZ1* and *JAZ6* [24]. Crucially, jasmonoyl-isoleucine (JA-Ile), but not other JA derivatives, mediates JAZ1-COI1 interaction in yeast two-hybrid and pull-down assays [24]. *CoI1-21* and *CoI1-22* are the missense alleles of *COI1* [25]. These missense mutations impair JA signaling resulting in JA–insensitivity. Besides, these mutants express two sets of disease resistance phenotypes. The first, also observed in coi1-1 null allele, includes enhanced basal defense against the virulent bacterial pathogen *Pto DC3000* and enhanced effector-triggered immunity (ETI) mediated by the NB–LRR RPM1 protein in both rar1 and wild-type backgrounds. These enhanced disease resistance phenotypes depend on the JA signaling function of *COI1*. Additionally, these mutants showed a unique inability to properly regulate *RPM1* accumulation and HR, exhibited increased *RPM1* levels in rar1, and weakened RPM1-mediated HR in *RAR1*. Importantly, there was no change in the steady-state levels or HR function of *RPM1* in coi1-1 [26]. In defense against necrotrophic pathogens, JA signaling activates defense-related genes like *PDF1.2* [27]. While *PDF1.2* and *THI2.1* are JA-inducible, their expression is non-responsive to JA in mapk4 mutants overexpressing *NaHG*, indicating the essential role of *MAPK4* in JA-responsive gene expression [14]. In bamboo, *PhJAZ1* shows higher expression in current-year leaves than in older ones, exhibiting a pattern opposite that of *PhSPL17*. *PhJAZ1* suppresses insect resistance via JA signaling, while PhSPL17 interacts with and negatively regulates PhJAZ1 [28]. Furthermore, in our previous studies, we systematically summarized the key genes involved in the SA and JA signaling pathways, as well as their regulatory relationships [29].

However, whether SBP-box genes in pepper participate in defense responses against *P. capsici* through SA and JA signaling pathways remains unreported. The SBP-box genes, a unique type of plant transcription factor first identified in *Antirrhinum majus* [30], is named for its ability to bind to the promoter region of the flower development gene *SQUAMOSA* and modulate its transcriptional activity [30]. Subsequent research has elucidated its critical role in plant ontogeny, morphogenesis, and adaptive responses to environmental stresses [31–35]. For instance, in tea plants (*Camellia sinensis* cv. Tie-guanyin), *CsSBP1*, *CsSBP17*, and *CsSBP19* all respond to light, shade, and cold stress, and are upregulated under these conditions [36]. In tomato, *SlSPL13* positively regulates inflorescence development by binding to the promoter of the flowering-related gene *SFT* [31]. Additionally, *TaSPL3* and *TaSPL17*, as targets of *miR156*, can regulate tillering and spikelet development by regulating the expression of *TaBA1* and *TaTB1* and interacting with the strigolactone (SL) signaling reporter TaD53 in wheat [37]. Similarly, the overexpression of *TaSPL20* and *TaSPL21* in wheat enhances inflorescence branching and promotes seed ontogeny [32]. In abiotic stress responses, *MdSPL13*, a target gene of *miR156a*, binds to the *MdWRKY100* promoter, thus enhancing salt tolerance in apple by upregulating *MdWRKY100* expression [33]. *OsSPL10* regulates rice drought tolerance by controlling the expression of its downstream gene *OsNAC2* and reactive oxygen species generation [34]. *ZaSPL21* from *Zanthoxylum armatum*, when overexpressed in *Nicotiana benthamiana*, can accelerate the germination rate of transgenic tobacco seeds under drought and salt stress conditions, and improve the salt tolerance of transgenic seedlings [38].

In biotic stress responses, phosphorylated *IPA1/OsSPL14* activates *WRKY45* expression, thereby enhancing rice blast resistance in rice plants [35]. Overexpression of *OsSPL4*, a target of Osa-miR535, also enhances blast resistance by upregulating defense genes and increasing hydrogen peroxide accumulation [39]. Furthermore, *AtSPL2*, *AtSPL10*, and *AtSPL11* in *Arabidopsis* regulate age-related resistance (ARR) in plants at adult stage. Notably, *AtSPL10*, targeted by miR156, mediates age-dependent enhancement of the salicylic acid (SA) pathway, potentially through direct activation of *PAD4*, a key component of systemic acquired resistance [40]. Intriguingly, OsMYB1 interacts with OsSPL14, but these transcription factors have antagonistic effects on the defense of rice against *brown planthopper* and *Xanthomonas oryzae* pv. *Oryzae*; *OsMYB1* acts as a negative regulator, whereas *OsSPL14* functions as a positive regulator [41].

In pepper, the SBP-box gene family comprises 15 members [42]. Previous studies have shown that *CaSBP12* and *CaSBP13* act as negative regulators in the response of pepper to salinity and drought stress, respectively [43, 44]. Additionally, *CaSBP08*, *CaSBP11*, and *CaSBP12* negatively regulate the response of pepper to *P. capsici* infection [29, 45, 46]. *CaSBP11* is hypothesized to modulatethe defense response of pepper against *P. capsici* by mediating the expression of defense-related genes (*CaBPR1*, *CaSAR8.2*, *CaDEF1*, and *CaPO1*) [29]. It has also been reported that *CaSAR8.2* participates in SA-mediated defense signaling pathways and serves as a molecular indicator for pathogen invasion in pepper [27]. *CaDEF1* is responsive to plant pathogen invasion and other abiotic stresses, as it is linked to JA-mediated signaling pathways [31]. *CaPO1* plays a role in regulating hydrogen peroxide levels and peroxidase activity in the hypersensitive response process associated with the interaction between pepper and nonhost pathogens [32]. However, whether the involvement of *CaSBP12* in the plant defense response is related to these defense-related genes is unknown. This study first found that the expression of these defense-related genes was induced in *CaSBP12*-silenced plants compared to control plants. Furthermore, the expression of these defense-related genes was inhibited in *CaSBP12*-overexpression pepper lines. Therefore, to further investigate the mechanism through which *CaSBP12* regulates the plant defense response to *P. capsici* inoculation, this study employed SA-signaling pathway mutants (sid2-2), JA-signaling pathway mutants (coi1-21 and coi1-22), and *Arabidopsis* plants overexpressing the salicylic acid hydroxamate gene (*NaHG*), which inhibits salicylic acid accumulation in plants. By analyzing the expression of genes associated with the SA and JA signaling pathways, this study preliminarily investigated the mechanism by which *CaSBP12* modulates plant defense response to *P. capsici* infection, establishing a critical foundation for further research into its regulatory role.

## Materials and methods

### Preparation of plant materials and pathogen

The *Capsicum annuum* pepper cultivar ‘A3’ and *P. capsici* strain HX-9 (belongings to physiological race ph3, to which ‘A3’ is susceptible) were obtained from the Pepper Research Group of the College of Horticulture and Landscape Architecture, Henan Institute of Science and Technology, Xinxiang, P. R. China. Columbia-0 ecotype *Arabidopsis thaliana* (Col-0) was obtained from previous laboratory (Vegetable Biotechnology and Germplasm Resources Innovation Laboratory, College of Horticulture, Northwest A&F University, Yangling, P. R. China.) propagation. The *Arabidopsis* mutant sid2-2 (Salk_111380), coi1-21 (cs68754), and coi1-22 (cs68755) were obtained from the SALK mutant library. Before using these mutants, homozygous mutants (sid2-2, coi1-21 and coi1-22) were screened for subsequent experiments. Detailed information on the screening of homozygous mutant sid2-2, coi1-21, and coi1-22 lines has been reported previously [47].

Pepper seeds were surface-sterilized with hot water treatment at 55 °C for 20 min, then planted in a plug tray with a ratio of peat, vermiculite, and perlite of 3:1:1, and subsequently cultivated in a controlled growth chamber starting January 3, 2018. During the day, the ambient temperature was maintained at 22 °C, while at night, the temperature was decreased to 18 °C. This consistent pattern was maintained by adhering to a strict 16-hour light cycle followed by an 8-hour dark period. Col-0 *Arabidopsis* and various mutant strains, including sid2-2, coi1-21, and coi1-22, were grown under identical experimental conditions as those used for pepper plants. The *P. capsici* strain HX-9 was propagated on potato dextrose agar (PDA) medium, composed of 200 g of potato, 17 g of agar, and 20 g of glucose per 1000 mL of water at 28 °C under dark conditions. (PDA was obtained from 200 g of potatoes cut into small pieces, which were boiled in sterile water for 20 min and then filtered through gauze. Then, 17 g of agar and 20 g of glucose were added, and the volume was increased to 1000 mL with water. The resulting PDA was then sterilized at 121 °C for 21 min.). Sporangium induction and zoospore release were executed following the protocol described by Wang et al. [48]. Briefly, after 5 days of growth on PDA, fungal colonies were cut into 0.8 cm diameter plugs using a cork borer. Ten plugs were placed into 9 cm Petri dishes containing 20 mL of liquid carrot medium (200 g of carrot per 1000 mL) and incubated at 28 °C in darkness for 3 days. The cultures were subsequently rinsed twice with sterile distilled water and incubated in 20 mL of salt solution (0.15 g of KH₂PO₄, 0.4 g Ca(NO₃)₂, 0.06 g of CaCl₂, and 0.15 g of Mg(NO₃)₂ per 1000 mL) at 28 °C for 5 days to induce sporangia formation. Zoospore release was triggered by cold shock (4 °C for 30 min) followed by 1.5 h of incubation at room temperature.

Zoospore concentration was standardized to 1 × 10⁵ spores/mL using a hemocytometer, as described by Jin et al. [49]. For root inoculation, 5 mL of the zoospore suspension (1 × 10⁵ spores/mL) was applied to pepper plants at the 6–8 true-leaf stage (approximately 75 days of seedling age) using the root-drench inoculation method [50]. Control plants received treatment with 5 mL of sterile water instead. Root tissue samples were collected at 0 and 2 days post-inoculation (dpi) and preserved at − 80 °C for further examination. For wild-type *Arabidopsis*, *CaSBP12* overexpression lines in *Arabidopsis*, *NaHG* overexpression lines in *Arabidopsis*, *CaSBP12* and *NaHG* co-expression lines in *Arabidopsis*, coi1-21, coi1-22, sid2-2 lines, and *CaSBP12* overexpression lines in these backgrounds, at 25-day seedling stage, root drenching inoculation method was used to inoculate with 5 mL of the zoospore suspension (1 × 10⁵ spores/mL) of *P. capsici*.

For detached leaf inoculation, the protocol outlined by Zhang et al. was followed [51]. Briefly, 10 leaves each from the 6th to 8th leaves from the bottom up of *CaSBP12*-silenced plants and control plants were collected (with each leaf obtained from a different plant) and placed in a tray containing filter paper pre-moistened with sterile water). Each leaf was inoculated with an 8-mm-diameter *P. capsici* mycelial disk and then incubated at 28 °C for 3 days.

### Silencing *CaSBP12* in pepper

The *CaSBP12* gene was silenced using a refined TRV-mediated virus-induced gene silencing (VIGS) technique, as described by Wang Jun’e [50]. The VIGS vector for *CaSBP12* silencing was constructed following the protocol outlined by Zhang et al. [46]. Recombinant vectors, including TRV2:*CaSBP12*, TRV2:*CaPDS* (phytoene desaturase, positive control), TRV2 (negative control), and TRV1, were introduced into *Agrobacterium tumefaciens* strain GV3101 via the freeze-thaw method and stored at −80 °C for subsequent use. For *CaSBP12* silencing, the VIGS procedure was performed on pepper plants at the two-true-leaf stage, following the method described by Zhang et al. [52]. After infiltration, the plants were incubated in darkness at 18 °C for 2 days and then transferred to a growth chamber with a 16/8 h light/dark photoperiod, a daytime temperature of 22 °C, a nighttime temperature of 18 °C, and 60% relative humidity. Once photobleaching was observed in the positive control plants (TRV2:*CaPDS*), detached leaves from the *CaSBP12*-silenced and control groups were collected to evaluate the effectiveness of *CaSBP12* gene silencing.

### Transient expression of *CaSBP12* in pepper

A transient expression vector for *CaSBP12* in pepper was developed (the vector was developed on January 4, 2016) following the protocol outlined by Zhang et al. [52]. *Agrobacterium tumefaciens* strain GV3101, harboring the pVBG2307:CaSBP12:GFP construct along with the pVBG2307:GFP construct (used as a control), was cultured overnight in Luria-Bertani (LB) medium supplemented with the appropriate antibiotics. The bacterial cells were then resuspended in an infiltration buffer consisting of 10 mM MgCl₂, 10 mM MES at pH 5.7, and 200 µM acetosyringone to prepare the inoculum. A cell suspension with an optical density of 0.8 was injected into the leaves of pepper plants (6–8 leaflets) using a needleless syringe [53]. Following injection, the plants were kept in the dark at 25 °C for 12 h, after which they were exposed to a light cycle of 16 h light and 8 h darkness at 70% humidity for 2 days. After this period, the injected leaves were collected and preserved at − 80 °C for further analysis.

### Overexpression of *CaSBP12* in *Arabidopsis Thaliana* and its mutants

The overexpression vector for the pepper *CaSBP12* gene in *Arabidopsis thaliana* and its mutants was constructed following the protocol described by Zhang et al. [52]. The *CaSBP12* gene was introduced into *Arabidopsis thaliana* and its mutants sid2-2 (Salk_111380), coi1-21 (cs68754), and coi1-22 (cs68755), using the floral dip transformation technique [54]. Transgenic strains exhibiting kanamycin resistance were thus obtained.

Additionally, the *NahG* gene encoding salicylate hydroxylase was isolated from *Pseudomonas putida* strain *ND6* and subcloned into the pVBG2307 vector on January 4, 2018. This gene was subsequently introduced into *Arabidopsis thaliana* via floral dip transformation, yielding kanamycin-resistant transgenic lines. Homozygous *NahG* transgenic lines were used as the maternal progenitor, while *CaSBP12* transgenic *Arabidopsis thaliana* lines served as the paternal progenitor in hybridization experiments, ultimately resulting in transgenic lines co-expressing both *NahG* and *CaSBP12* genes.

The primers used for vector construction and identification of *Arabidopsis thaliana* mutant strains (including sid2-2, coi1-21, and coi1-22) are detailed in Supplementary Table 1.

### Disease index assessment

The disease index was evaluated following the protocol established by Zhang Yingli [55]. In this experiment, 16 days post-inoculation with *P. capsici* strain HX-9, the disease severity in *CaSBP12*-silenced and control plants was categorized into four distinct levels based on symptom observations. The severity levels are defined as follows: level 0: absence of disease symptoms; level 1, abscission of lower and middle leaves, with noticeable constriction at the root-stem junction. level 2, entire plant exhibits wilting, lower leaves have abscised, and constriction is evident at both the root-stem junction and stem internodes; level 3, death of all plants components except the apical meristem, or complete plant mortality. The number of diseased plants at each level in both CaSBP12-silenced and control groups was counted. The disease index percentage of the plants was calculated using the methodology outlined by Zhang Yingli [55]. This experiment was repeated three times, with 50 *CaSBP12*-silenced plants and 50 control plants in each replicate.

### RNA extraction and quantitative real-time PCR

RNA was isolated using the TaKaRa MiniBEST Universal RNA Extraction Kit, and cDNA was synthesized from the isolated RNA using the PrimeScript™ RT reagent kit (Takara, Dalian, China), following the manufacturer’s instructions. The resulting cDNA was diluted to a concentration of 50 ng/µL for quantitative real-time PCR (qRT-PCR). The qRT-PCR was performed using the iCycler iQ™ Multicolor PCR Detection System (Bio-Rad, Hercules, CA, USA) under the following thermal cycling conditions: initial denaturation at 95 °C for 1 min, followed by 40 cycles of denaturation at 95 °C for 10 s, annealing at 56 °C for 30 s, and extension at 72 °C for 30 s. The primer sequences employed for qRT-PCR are detailed in Supplementary Table 2, with their specificity validated via the NCBI Primer-BLAST tool (http://www.ncbi.nlm.nih.gov/tools/primer-blast/index.cgi?LINK_LOC=BlastHome). The pepper actin gene (*CaActin2*, GenBank accession number: AY572427) and the *Arabidopsis thaliana* actin gene **(***AtActin8*, AT1G49240) served as the internal control [56]. The relative quantification of target gene expression was calculated using the 2^-ΔΔCt^ method as described by Schmittgen and Livak [57].

### Statistical analysis

A comprehensive statistical analysis was executed employing the SPSS 22.0 (IBM Corp., Armonk, NY, USA). Variation among treatments was assessed via one-way ANOVA, with significant differences among treatment means determined through Tukey’s post hoc test at significance levels of *P <* 0.05 and *P <* 0.01. The results are reported in the form of mean values ± standard deviation (SD). In all experimental procedures, we evaluated a minimum of three independent biological samples, which were meticulously analyzed following a precisely structured methodology to ensure reproducibility and statistical validity.

## Results

### Silencing of *CaSBP12* enhances plant resistance to *P. capsici* infection

**Fig. 1:**
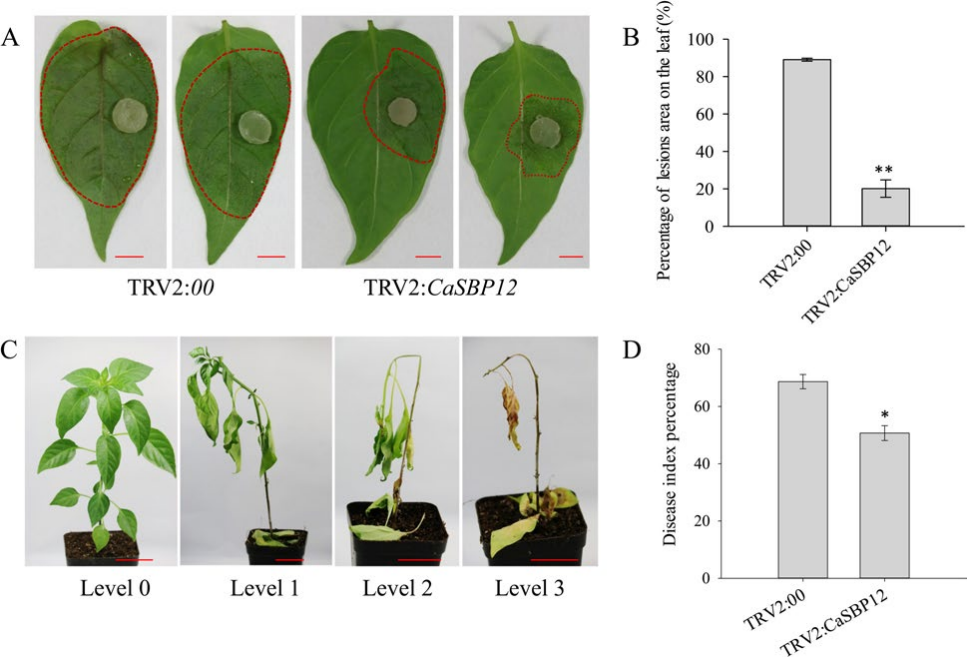
Evaluation of disease resistance in *CaSBP12*-silenced pepper plants. (**a**) Morphological assessment of detached leaves from *CaSBP12-*silenced and control plants three days post-inoculation with* P. capsici*. The area circled in red indicates water-soaked lesions. Scale bar, =4 mm. (**b**) Quantification of lesion area percentage in leaves of *CaSBP12*-silenced versus control plants 3 days post-inoculation with *P. capsici*. (**c**) Disease index grading criteria for *CaSBP12*-silenced and control plants sixteen days post-inoculation with* P. capsici*. Scale bar=3.5 cm. (**d**) Disease index percentage for *CaSBP12*-silenced and control plants sixteen days post-inoculation with *P. capsici*. Variation associated with treatment was evaluated through one-way ANOVA, and significant differences among treatment groups were evaluated at significance levels of *P* < 0.05 and* P* < 0.01 using Tukey’s post hoc test; * and ** indicate significant differences at *P *<0.05 and *P *<0.01, respectively. Mean values and standard deviations for three biological replicates are presented

**Fig. 2:**
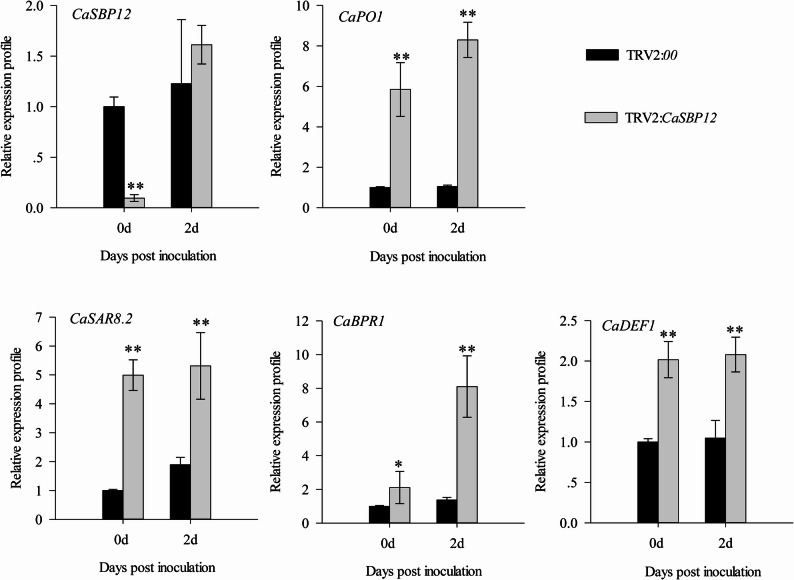
Expression levels of defense-related genes post-inoculation with *P. capsici *in *CaSBP12*-silenced and control plants. Variation among treatment groups was evaluated through one-way ANOVA, and significant differences among treatment groups were evaluated at significance levels of *P* < 0.05 and *P* < 0.01 using Tukey’s post hoc test; * and ** denote significant differences at *P *< 0.05 and *P *< 0.01, respectively. Mean values and standard deviations for three biological replicates are shown

In this study, virus-induced gene silencing (VIGS) technology was used to silence the *CaSBP12* gene in pepper, achieving a silencing efficiency of 75% (Supplementary Fig. 1). Without stress, no notable phenotypic differences were observed between *CaSBP12*-silenced (TRV2:*CaSBP12*) plants and control (TRV2:*00)* plants (Supplementary Fig. 1). Three days post-inoculation with *P. capsici*, both groups exhibited water-soaked lesions on detached leaves (Fig. 1a), but the lesion area was significantly smaller in the *CaSBP12*-silenced plants (Fig. 1b). Sixteen days post-inoculation, the infected plants were categorized into four severity levels based on symptomatology. Level 0 indicated asymptomatic plants; level 1 was characterized by abscission of lower leaves with constriction at the root-stem junction; level 2 involved complete wilting of the plant alongside lower leaf abscission and constriction between the roots and stem; level 3 indicated either the death of the plant aside from the growing point or total plant death (Fig. 1c). Moreover, statistical analysis revealed that the disease index percentage for the *CaSBP12*-silenced plants was significantly lower than that of the control group (Fig. 1d). Additionally, the expression of defense-related genes in both groups was measured at both 0 and 2 days post-inoculation. The expression of defense-related genes (*CaSAR8.2*, *CaDEF1 CaPO1*, and *CaBPR1*,) was significantly elevated in *CaSBP12*-silenced plants compared to controls, at 0 and 2 days post-inoculation (Fig. 2). These findings suggest that silencing *CaSBP12* enhances the disease resistance of pepper plants.

### Transient expression of *CaSBP12* in pepper

**Fig. 3:**
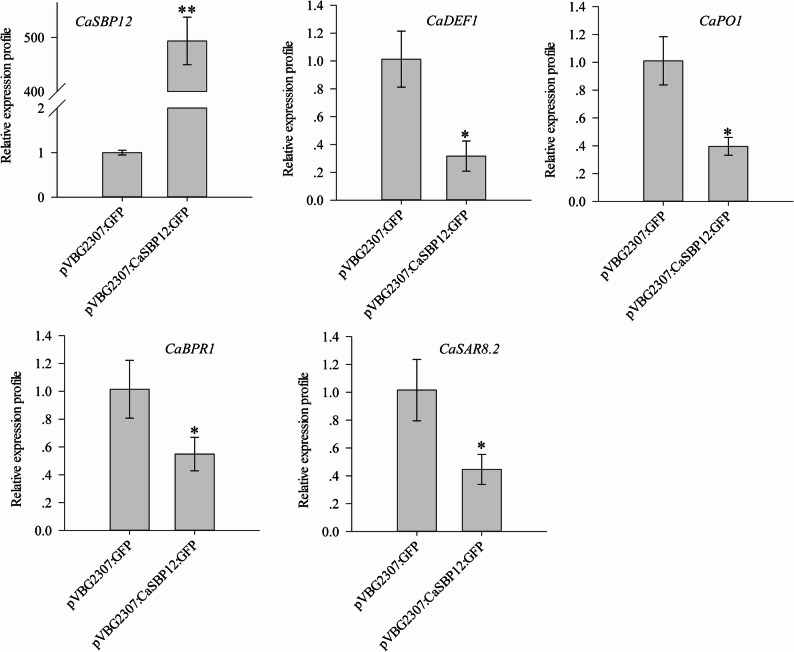
Expression levels of defense-related genes under *CaSBP12* transient expression in pepper plants. Here, pVBG2307: CaSBP12: GFP represents *CaSBP12* transient expression plants, and pVBG2307: GFP represents control plants. Variation among treatments was evaluated through one-way ANOVA, and significant differences among treatment groups were distinguished at significance levels of *P* < 0.05 and *P* < 0.01 using Tukey’s post hoc test; * and ** signify significant differences at thresholds of* P* < 0.05 and *P* < 0.01, respectively. Mean values and standard deviations for three biological replicates are presented

The above experiment demonstrated that silencing *CaSBP12* increased expression of the defense-related genes *CaSAR8.2*, *CaDEF1*, *CaPO1*, and *CaBPR1*, even in the absence of pathogen treatment. This suggests that *CaSBP12* may inhibit the expression of these defense-related genes. To investigate this further, *CaSBP12* was transiently expressed in pepper plants (Fig. 3). As shown in Fig. 3, the transient expression of *CaSBP12* in pepper plants (pVBG2307:CaSBP12:GFP) significantly exceeded that of the control group (pVBG2307:GFP). Additionally, the defense-related genes *CaDEF1*, *CaSAR8.2*, *CaPO1*, and *CaBPR1* showed marked suppression in plants with transient *CaSBP12* expression.

### Overexpression of the pepper *CaSBP12* in *Arabidopsis Thaliana* and its mutants

To further elucidate the mechanistic role of *CaSBP12* in plant defense against *P. capsici*, this experiment incorporated mutants with impaired SA signaling (*SID2-2*, Salk_111380) and JA signaling (*COI1-21*, cs68754; *COI1-22*, cs68755) [25, 26, 58].

Additionally, this experiment has involved the overexpression of the SA hydroxamic acid gene (*NaHG*), which prevents SA accumulation, in *Arabidopsis thaliana*. The detection of homozygotes of the introduced mutations refers to the study of Zhang in 2020 [47].

**Fig. 4:**
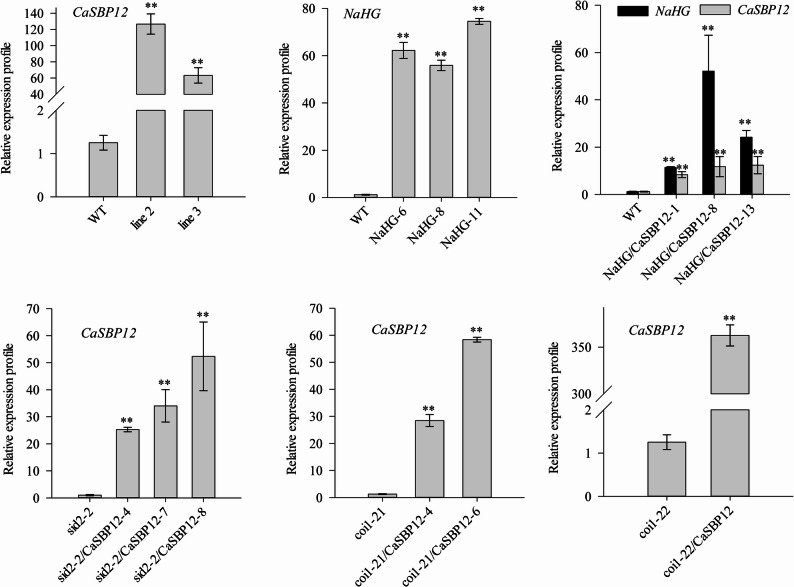
Overexpression of *CaSBP12* in *Arabidopsis*, overexpression of SA hydroxamic acid gene (*NaHG*) in *Arabidopsis*, and the expression levels of *CaSBP12* in sid2-2, coi1-21, coi1-22, and *NaHG Arabidopsis* overexpression lines. Line 2 and Line 3 represent the *CaSBP12*-overexpression lines in *Arabidopsis*; NaHG-6, NaHG-8, and NaHG-11 represent the *NaHG*-overexpression lines in *Arabidopsis*; NaHG/CaSBP12-1, NaHG/CaSBP12-8, and NaHG/CaSBP12-13 represent the hybrid lines overexpressing *NaHG* and *CaSBP12* in *Arabidopsis*; sid2-2/CaSBP12-4, sid2-2/CaSBP12-7, and sid2-2/CaSBP12-8 represent the *CaSBP12-*overexpression lines in the sid2-2 background; coi1-21/CaSBP12-4 and coi1-21/CaSBP12-6 represent *CaSBP12-*overexpression lines in the coi1-21 background; coi1-22/CaSBP12 represents the *CaSBP12-*overexpression lines in the coi1-22 background. ** denotes significant differences at a threshold of *P* < 0.01. Mean values and standard deviations for three biological replicates are shown

For subsequent experiments, defined transgenic lines *CaSBP12* (line 2 and line 3) and 3 *NaHG* (line 6, line 8, and line 11) were used. As shown in Fig. 4, the transcriptional levels of *CaSBP12* and *NaHG* in the transgenic lines were markedly elevated compared to the wild-type (WT) plants. Furthermore, the hybrid lines containing both *CaSBP12* and *NaHG* also exhibited significantly higher expression levels than the WT plants. Moreover, *CaSBP12* was successfully overexpressed in the coi1-21, coi1-22, and sid2-2 backgrounds (Fig. 4).

**Fig. 5:**
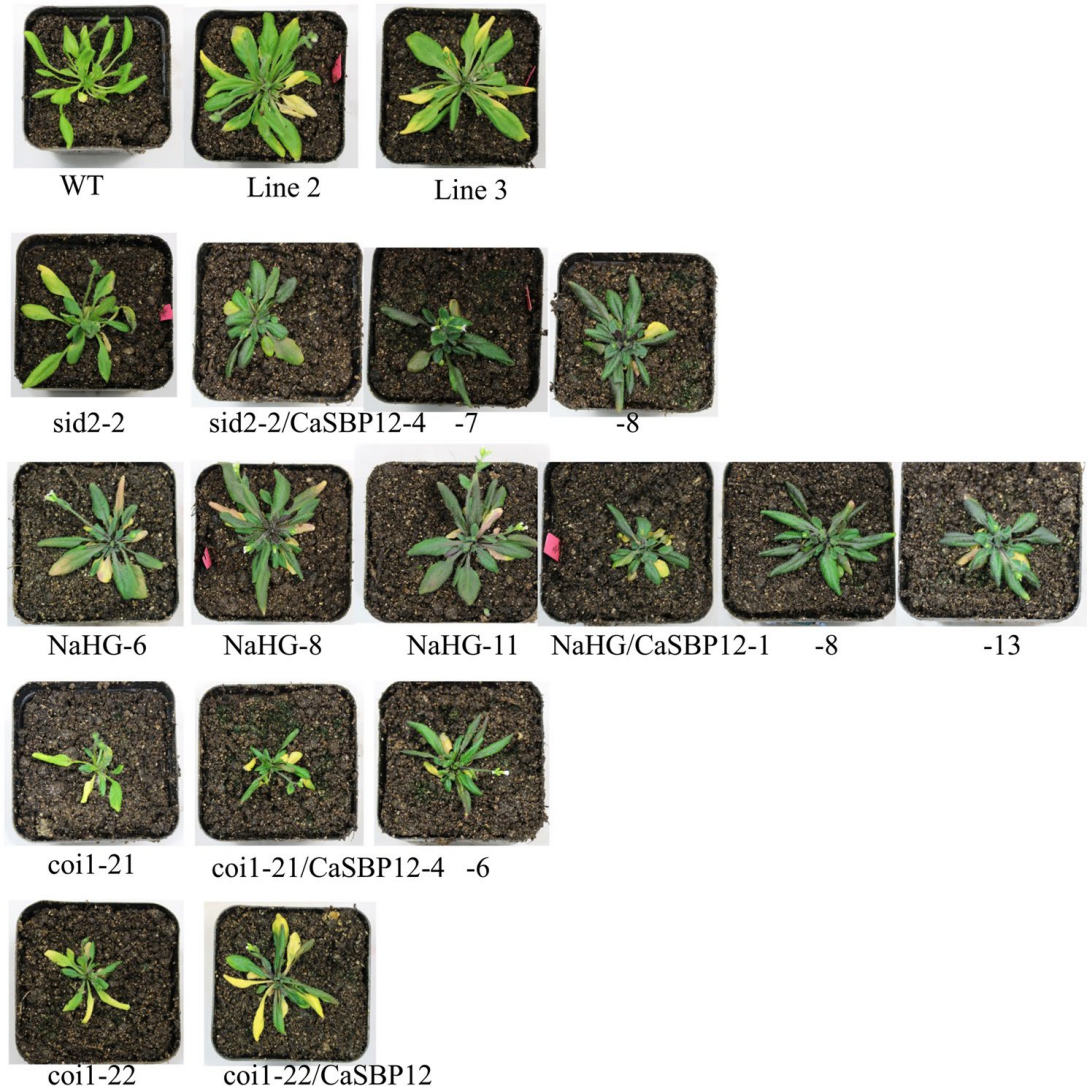
Phenotypic characteristics of transgenic lines observed three days post-inoculation with *P. capsici*. Line 2 and Line 3 represent the overexpression lines of *CaSBP12* in *Arabidopsis*; sid2-2/CaSBP12-4, −7, −8 represent the overexpression lines of *CaSBP12 *in sid2-2; NaHG-6, NaHG-8, NaHG-11 represent the overexpression lines of *NaHG* in *Arabidopsis*; NaHG/CaSBP12-1, −8, −13 represent the hybrid lines after overexpressing *NaHG* and *CaSBP12* in *Arabidopsis*, respectively; coi1-21/CaSBP12-4, −6 represent the overexpression lines of *CaSBP12* in coi1-21; coi1-22/CaSBP12 represents the overexpression line of *CaSBP12* in coi1-22

Additionally, after inoculating with *P. capsici* three days, different strains exhibit various symptoms of the disease; some strains show differing degrees of chlorosis in the lower leaves, while others have deeper-colored leaves. However, overall, the sid2-2, coi1-21, coi1-22, and *NaHG-*overexpression lines are more susceptible than the wild-type plants, while the susceptibility of the *NaHG* and *CaSBP12* hybrid lines is phenotypically lower than that of the *NaHG* and *CaSBP12* overexpression lines respectively (Fig. 5). Similarly, the susceptibility of *CaSBP12* overexpression lines in the sid2-2 mutant is lower than that of the sid2-2 mutant and *CaSBP12 Arabidopsis* overexpression lines (Fig. 5). However, the susceptibility of *CaSBP12* overexpression lines in the coi1-21 and coi1-22 mutants is slightly more severe than that of the corresponding coi1-21 and coi1-22 mutants (Fig. 5).

**Fig. 6:**
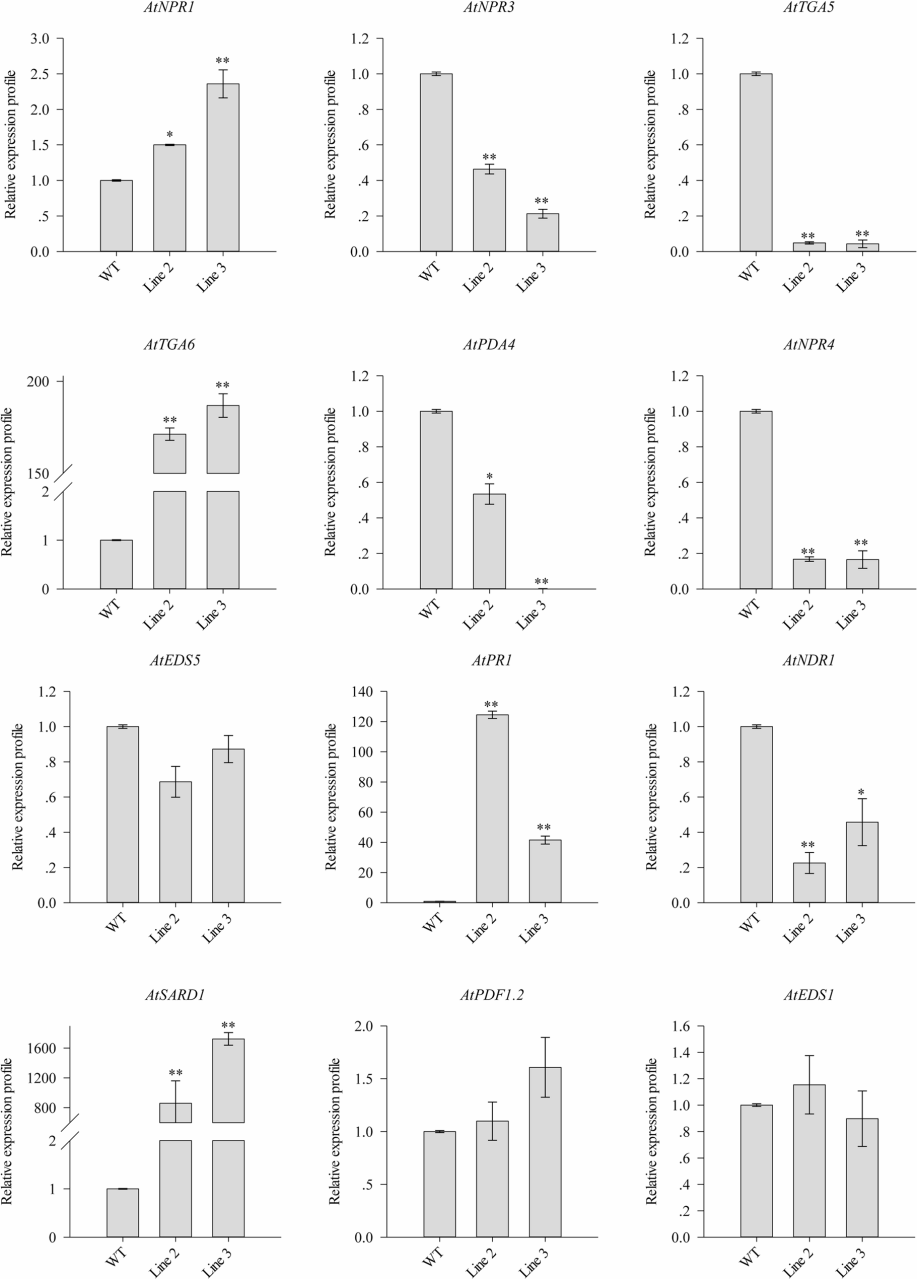
Expression levels of genes associated with the salicylic acid and jasmonate signaling pathways in *CaSBP12 *transgenic *Arabidopsis* lines. Line 2 and Line 3 represent the *CaSBP12*–overexpression lines in *Arabidopsis*. Bars labelled with different letters indicate significant differences at a threshold of *P* < 0.05 according to Tukey’s post hoc test. Mean values and standard deviations for three replicates are displayed

**Fig. 7:**
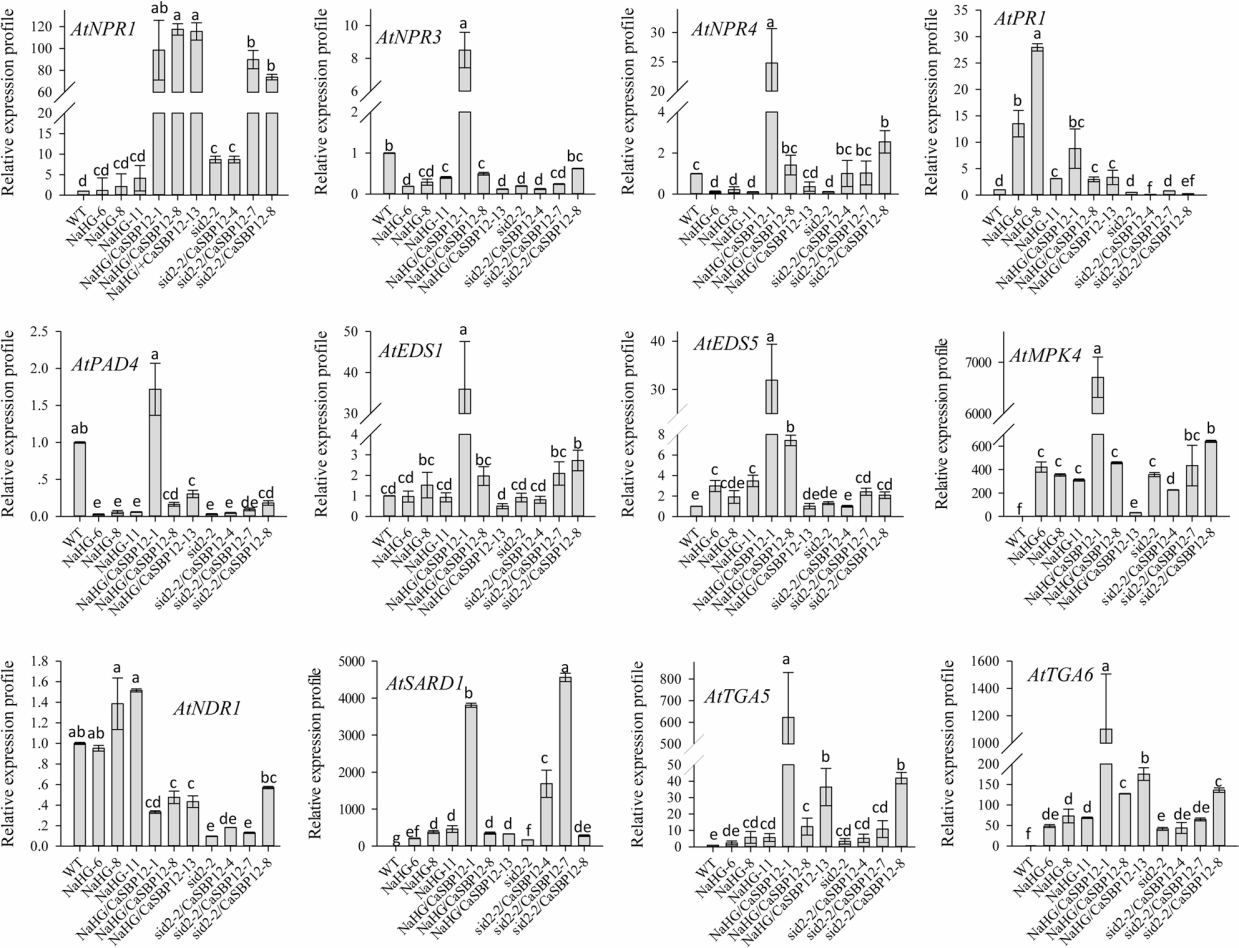
Expression of salicylic acid pathway-related genes in *NaHG*-overexpression lines, *NaHG* and *CaSBP12* co-expressing lines, sid2-2 lines, and *CaSBP12*-overexpression lines in the sid2-2 background. NaHG-6, NaHG-8, and NaHG-11 represent the *NaHG*-overexpression lines in *Arabidopsis*; NaHG/CaSBP12-1, NaHG/CaSBP12-8, and NaHG/CaSBP12-13 represent the hybrid lines overexpressing *NaHG *and *CaSBP12* in *Arabidopsis*; sid2-2/CaSBP12-4, sid2-2/CaSBP12-7, and sid2-2/CaSBP12-8 represent the *CaSBP12-*overexpression lines in the sid2-2 background. Bars labelled with different letters denote significant differences at a threshold of *P* < 0.05 according to Tukey’s post hoc test. Mean values and standard deviations for three replicates are shown

Besides, this study evaluated gene expression levels associated with the SA and JA signaling pathways in *CaSBP12* transgenic lines. Genes related to the SA pathway, such as *AtNPR1*, *AtPR1*, *AtTGA6*, and *AtSARD1*, showed increased expression, while *AtNPR3*, *AtNDR1*, *AtNPR4*, *AtPAD4*, and *AtTGA5* exhibited reduced expression (Fig. 6). Conversely, the transcription levels of *AtEDS1*, *AtEDS5*, and *AtTGA4* showed no significant deviation from those observed in WT plants (Fig. 6, Supplementary Fig. 2). Regarding the JA signaling pathway, the expression of *AtPDF1.2* was notably increased in the *CaSBP12* transgenic lines (Fig. 6). Additionally, the transcript levels of genes linked to the ethylene signaling pathway were also evaluated. As depicted in Supplementary Fig. 2, the transcription level of *AtETR1* was diminished in the *CaSBP12* transgenic lines. Furthermore, this study assessed the expression profiles of genes associated with the SA signaling pathway in *NaHG*-overexpression lines, *NaHG* and *CaSBP12* co-expression lines, sid2-2 lines, and *CaSBP12-*overexpression lines within the sid2-2 background. In *NaHG*-overexpression lines, *AtNPR1*, *AtPR1*, *AtEDS5*, *AtMPK4*, *AtNDR1*, *AtSARD1*, *AtTGA5*, and *AtTGA6* were upregulated (Fig. 7). Conversely, *AtNPR3*, *AtPAD4*, *AtNPR4*, and *AtTGA4* showed decreased expression (Fig. 7, Supplementary Fig. 3). The expression levels of *AtNPR1*, *AtNDR1*, *AtEDS1*, and *AtTGA2* did not differ significantly from those of WT plants (Fig. 7, Supplementary Fig. 3). Notably, in the *NaHG* and *CaSBP12* co-expression lines, the expression levels of *AtEDS1*, *AtNPR3*, *AtPAD4*, *AtSARD1*, *AtTGA5*, *AtEDS5*, *AtMPK4*, *AtNPR4*, *AtTGA6*, *AtTGA2*, *AtNPR1*, and *AtTGA4* surpassed those in the *NaHG*-overexpression lines (Fig. 7, Supplementary Fig. 3). However, the expression level of *AtPR1*, *AtNDR1*, and *AtCBP60g* were lower in the co-expression lines compared to the *NaHG*-overexpression lines (Fig. 7, Supplementary Fig. 3). In sid2-2 lines, expression levels of *AtNPR1*, *AtTGA5*, *AtMPK4*, *AtSARD1*, and *AtTGA6* were upregulated relative to WT plants, while *AtNPR3*, *AtNDR1*, *AtNPR4*, *AtPAD4*, and *AtTGA4* were downregulated (Fig. 7, Supplementary Fig. 3). Furthermore, the expression levels of *AtEDS1*, *AtCBP60g*, *AtTGA2*, *AtPR1*, and *AtEDS5* in sid2-2 lines remained comparable to those of the WT plants (Fig. 7, Supplementary Fig. 3). In *CaSBP12-*overexpression lines within the sid2-2 background, *AtPR1* expression was decreased in comparison to those in the control sid2-2 lines (Fig. 7). Conversely, the expression levels of *AtPAD4*, *AtEDS1*, *AtMPK4*, *AtNDR1*, *AtNPR1*, *AtSARD1*, *AtNPR3*, *AtTGA5*, *AtEDS5*, *AtTGA6*, *AtNPR4*, *AtTGA2*, and *AtCBP60g* were higher in *CaSBP12*-overexpression lines compared to the control sid2-2 lines (Fig. 7, Supplementary Fig. 3).

**Fig. 8:**
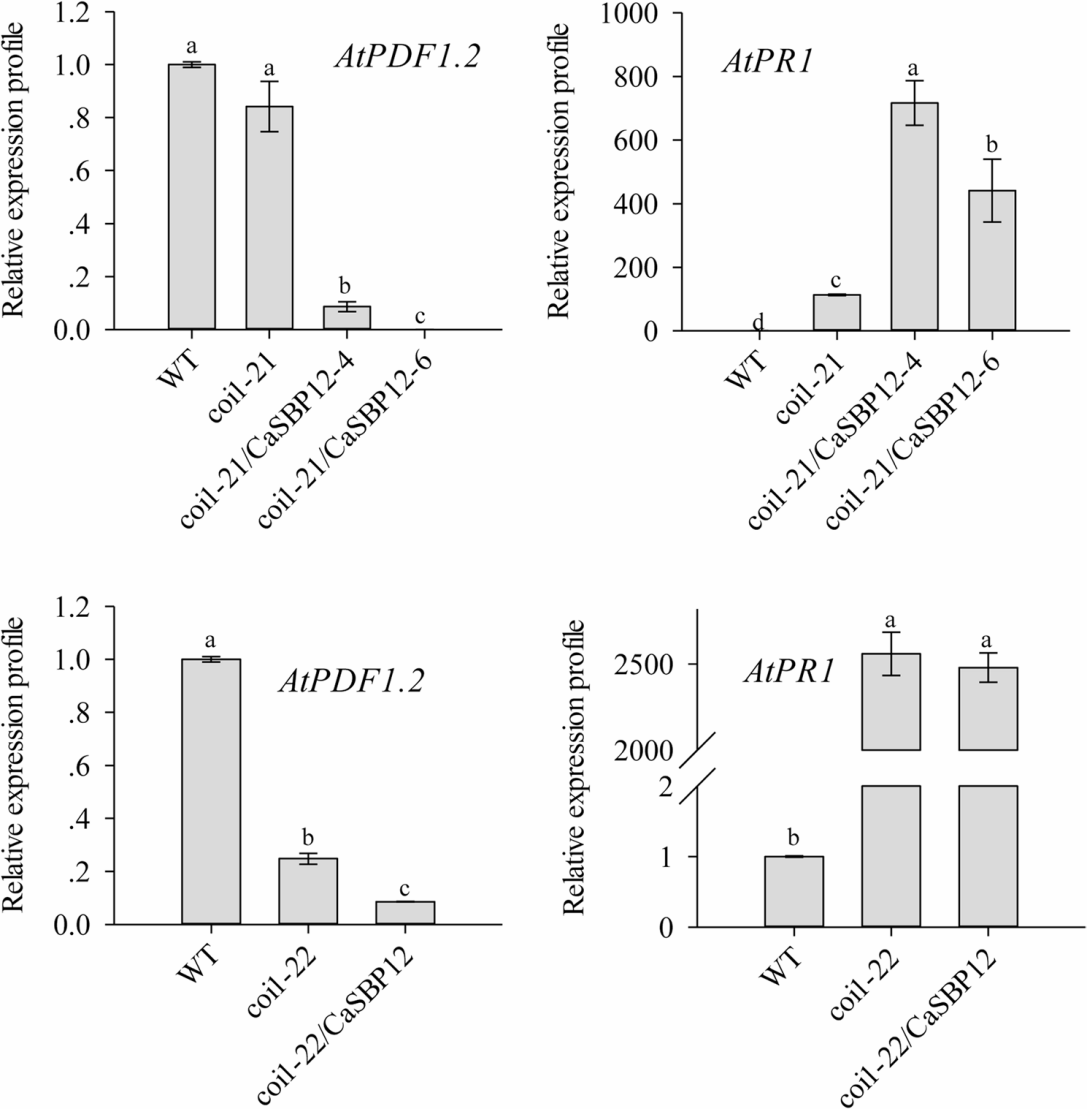
Expression levels of genes related to the jasmonic acid signaling pathway in coi1-21 and coi1-22 backgrounds, and lines overexpressing *CaSBP12* in coi1-21 and coi1-22 backgrounds. coi1-21/CaSBP12-4 and coi1-21/CaSBP12-6 represent the CaSBP12-overexpression lines in the coi1-21 background; coi1-22/CaSBP12 represents the CaSBP12-overexpression line in the coi1-22 background. Bars labelled with different letters indicate significant differences at a threshold of* P* < 0.05 according to Tukey’s post hoc test. Mean values and standard deviations for three replicates are shown

Additionally, this study examined the expression of JA signaling pathway genes in the coi1-21 and coi1-22 backgrounds, as well as in the *CaSBP12*-overexpression lines in these same backgrounds. Figure 7 illustrates that *AtPDF1.2* expression was reduced in the coi1-22 background compared to the WT background. Conversely, *AtPR1* expression was elevated in both coi1-21 and coi1-22 backgrounds relative to the WT background (Fig. 8). Moreover, in the *CaSBP12*-overexpression lines in the coi1-21 and coi1-22 backgrounds, the *AtPDF1.2* expression level was lower relative to the respective coi1-21 and coi1-22 background controls (Fig. 8). Simultaneously, *AtPR1* expression was enhanced in the *CaSBP12*-overexpressing coi1-21 background controls compared to the coi1-21 background (Fig. 8). However, under *CaSBP12*-overexpression in the coi1-22 background, there was negligible change in *AtPR1* expression compared to the coi1-22 background (Fig. 8).

## Discussion

The SBP-box gene family, unique to plants, functions as transcription factors crucial for plant growth and development, signal transduction, and various stress responses [34, 36, 40]. Previous research has identified 15 SBP-box genes in pepper, with *CaSBP08*, *CaSBP11*, and *CaSBP12* acting as negative regulators in the plant defense mechanism against *P. capsici* infection [29, 45, 46]. Notably, *CaSBP11* is involved in the plant defense response though regulating genes associated with this process [29]. Nonetheless, the role of *CaSBP12* in the defense response against *P. capsici* infection is not yet fully elucidated. Based on previous research, this study further found that *CaSBP12* may participate in the defense response of plants to pepper *Phytophthora* blight infection through the SA signaling pathway.

Previous research has shown that the *CaSBP12* gene consists of 900 base pairs, encoding 299 amino acids, with a nuclear localization signal and two (C3H and C2HC) zinc finger structures [42]. Moreover, the CaSBP12 protein localizes within the nucleus [46]. Silencing of *CaSBP12* enhanced resistance to *P. capsici* infection (Fig. 1). According to a previous report, *CaSAR8.2* (Systemic acquired resistance gene)participates in SA-mediated defense signaling pathways and serves as a molecular indicator for pathogen invasion in pepper [59]. Similarly, in tobacco, the *SAR8.2* gene (Systemic acquired resistance gene) enhances resistance to *P. nicotianae* infection [60]. Additionally, in pepper, the *CaBPR1* promoter plays a critical role in modulating the expression of *CaBPR1* gene (Pepper pathogenesis-related (PR)−1 protein) in reaction to biotic and abiotic stressors as well as environmental challenges [61]. Moreover, the elevated expression of *CaBPR1* in *Nicotiana tabacum* conferred increased resistance to the oomycete pathogen *Phytophthora nicotianae* [62]. *CaDEF1* (Pepper defensin gene) is responsive to plant pathogen invasion and other abiotic stresses, as it is linked to JA-mediated signaling pathways [63]. *CaPO1*(Pepper peroxidase-like gene) plays a role in regulating hydrogen peroxide levels and peroxidase activity in the hypersensitive response process associated with the interaction between pepper and nonhost pathogens [64]. Therefore, this study assayed the expression of these genes in *CaSBP12*-silenced and control pepper plants post-inoculation with *P. capsici*. As depicted in Fig. 2, two days post-inoculation with *P. capsici*, the expression of these genes in *CaSBP12*-silenced plants was markedly elevated compared to the control plants. Notably, even at zero days post-inoculation, these genes were more highly expressed in *CaSBP12*-silenced plants. Thus, it was hypothesized that *CaSBP12* potentially augments the resistance of pepper plants to *P. capsici* infection through modulating the expression of these defense-related genes. To test this hypothesis, transient expression of the *CaSBP12* gene was conducted in pepper plants. Subsequent quantification of defense-related gene expression levels in the *CaSBP12*-transiently expressed lines revealed marked repression of *CaSAR8.2*, *CaBPR1*, *CaDEF1*, and *CaPO1* expression (Fig. 3). These findings imply that *CaSBP12* may play a role in the pepper defense mechanisms against *P. capsici* by modulating the transcriptional level of these defense-related genes. Nonetheless, it remains uncertain if *CaSBP12* is directly involved in the specific signaling pathways regulating these genes. Previous research has documented that the pathogen-responsive marker gene *CaSAR8.2* (*CASAR82A*) can be activated by SA at concentrations of 0.01, 0.1, 1, 2, and 5 mM, as well as methyl jasmonate (at 0.1, 1, 10, 50, and 100 µM) [59]. Additionally, it participates in the SA-dependent disease resistance signaling cascade [59, 65]. The expression level of *CaBPR1* in pepper can be activated by nitric oxide (100 µM), salicylic acid (5 mM or 1 mM), ethylene (1 mM), or methyl jasmonate (56 µM or 50 µM), as well as biotic and abiotic stresses [61, 66]. Furthermore, the expression of the JA-responsive gene *CaDEF1* is upregulated by SA (0.01, 0.1, 1, 2 and 5 mM) and is involved in the signaling pathways that are dependent on JA [63, 67]. Similarly, the transcription of *CaPO1* can be stimulated by SA (5 mM) [64].

**Fig. 9:**
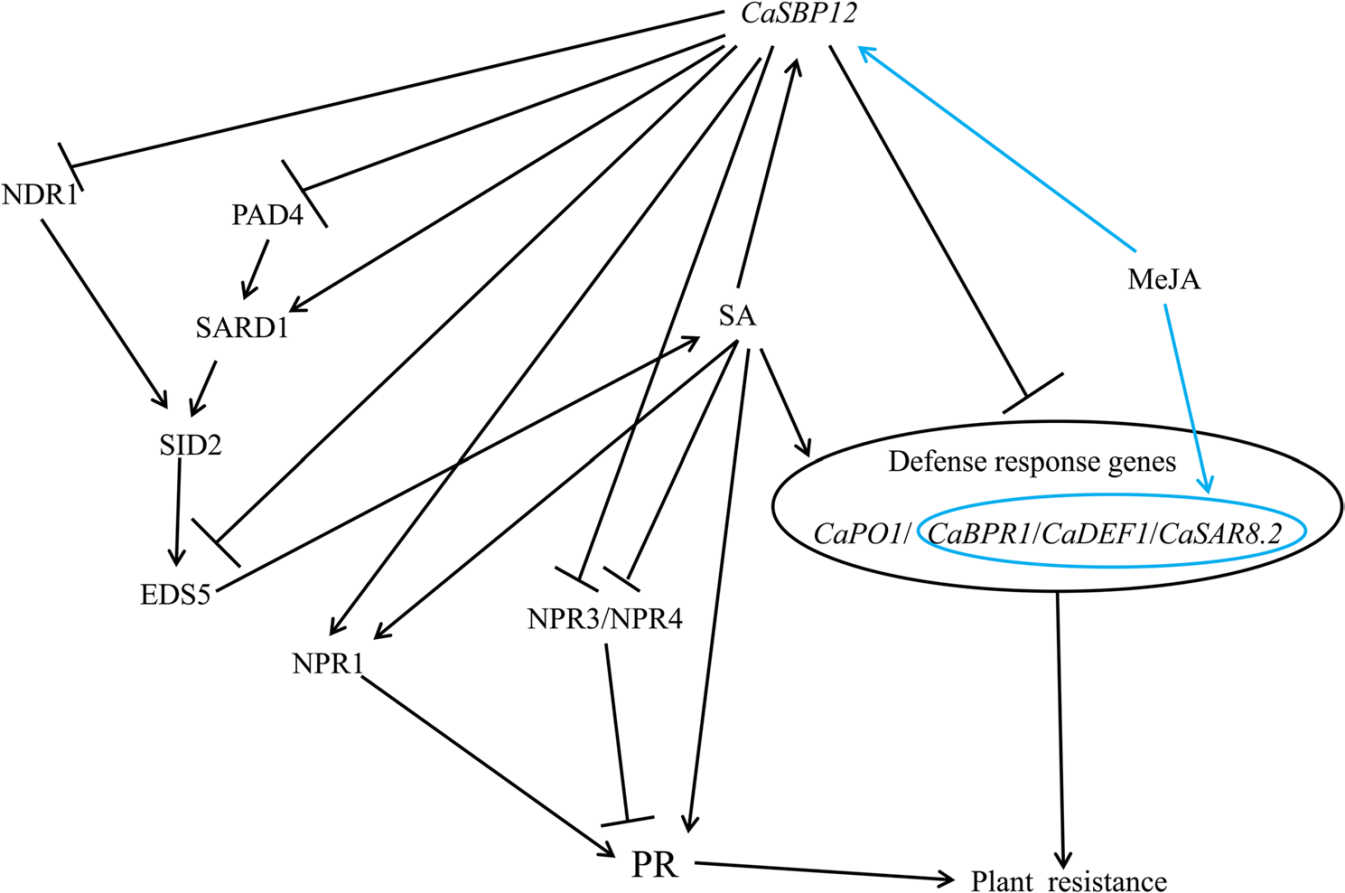
Proposed model for *CaSBP12 *involvement in plant defense mechanisms. Arrows represent positive regulation, while the absence of an arrow denotes negative regulation. The blue line indicates that MeJA promotes the expression of the *CaSBP12* gene and the defense-related genes *CaBPR1*,*CaDEF1*, and *CaSAR8.2*. *CaPO1*, Pepper peroxidase-like gene; *CaBPR1*, Pepper pathogenesis-related (PR)−1 protein; *CaDEF1*, Pepper defensin gene; *CaSAR8.2*, Systemic acquired resistance gene; *NDR1*, nonrace specific disease resistance 1;* PAD4*, Phytoalexin deficient 4; *SARD1*, systemic acquired resistance deficient 1; *SID2-2*, Salicylic acid induction deficient 2; *EDS5*, MATE efflux family protein; *NPR1*, nonexpressor of PR 1; *NPR3*, NPR1-like protein 3; *NPR4*, NPR1-like protein 4; *PR*, Pathogenesis-related gene

There are key limitations in research resulting from the challenge of translating gene function knowledge across the substantial evolutionary distance between *Arabidopsis* and various crop species, such as pepper, without comparative genomics support [68]. However, our previous research indicates that *CaSBP12* is evolutionarily closely related to *AtSPL8* in *Arabidopsis* [42]. Additionally, advancements in genomic analysis techniques have revealed a high degree of conservation in molecular mechanisms among even evolutionarily distant plant species [68]. Furthermore, researchers in our laboratory have also successfully verified the function of pepper *CaHsp25.9* previously in *Arabidopsis* under drought, salt, and heat stress conditions [69]. Therefore, to further elucidate the role of the *CaSBP12* gene in the SA and JA signaling pathways, the present study involved the overexpression of *CaSBP12* in *Arabidopsis thaliana* and some of its various mutants deficient in elements of the SA synthesis pathway (sid2-2) and JA synthesis pathway (coi1-21 and coi1-22). Additionally, co-expression of *CaSBP12* with the *NaHG* gene, which inhibits SA accumulation, was conducted in *Arabidopsis thaliana* (Fig. 4). After inoculation with *P. capsici*, the sid2-2, coi1-21, coi1-22, and NaHG lines exhibited greater susceptibility compared to the wild-type plants (Fig. 5). In contrast, the susceptibility of the *NaHG* and *CaSBP12* hybrid lines was phenotypically lower than that of the *NaHG* and *CaSBP12* overexpression lines, respectively (Fig. 5). Similarly, the susceptibility of the *CaSBP12* overexpression lines in the sid2-2 mutant was lower than that of the sid2-2 mutant and the *CaSBP12 Arabidopsis* overexpression lines (Fig. 5). However, the susceptibility of the *CaSBP12* overexpression lines in the coi1-21 and coi1-22 mutants was slightly more severe than that of the corresponding coi1-21 and coi1-22 mutants (Fig. 5). We speculate that this phenomenon may be attributed to the ability of salicylic acid (SA) to promote the expression of the *CaSBP12* gene, which serves as a negative regulatory gene [42, 46]. When SA is present, plants tend to be more susceptible. Conversely, in the absence of SA, the expression level of the *CaSBP12* gene decreases, resulting in increased resistance. In the *CaSBP12* overexpression lines of coi1-21 and coi1-22, the expression level of *AtPDF1.2* decreased, while the expression levels of *AtPR* genes increased (Fig. 8). However, Fig. 8 shows significant expression differences of the gene among coi1 mutants with different genetic backgrounds, which may be due to the presence of both functionally similar and different parts in the various *COI1* alleles. For instance, the *COI1* alleles coi-21, coi1-22, coi1-1, and coi1-16 exhibit enhanced disease resistance against *Pto DC3000 (EV)* under the rar1 and *RAR1* backgrounds, but they have different effects on *RPM1* accumulation and the *RPM1*-mediated immune response. coi1-1 does not alter *RPM1* levels, coi1-16 enhances *RPM1* levels in rar1 but has no effect on the *RPM1*-mediated HR in RAR1, while coi1-21 and coi1-22 enhance *RPM1* levels in rar1 but reduce the *RPM1*-mediated HR in *RAR1* [25]. Besides, Figs. 6 and 7, and 8 demonstrate that *CaSBP12* modulates the expression of genes within the SA and JA signaling pathways to varying degrees. In the SA-mediated signaling pathway, genes that include *PAD4*, *NDR1*, *NPR1*, *SID2*, *EDS5*, and *EDS1* positively regulate the defense of *Arabidopsis thaliana* against *P. capsici* infestans [70]. Except for *NPR1*, these genes are upstream in the SA signaling cascade [29]. Overexpression of *NaHG* in *Arabidopsis thaliana* inhabits SA accumulation and alters the expression of associated genes in the SA pathway (Fig. 7, Supplementary Fig. 3). It has been reported that the major positive immune regulators*CBP60g* and *SARD1*, function between the SA biosynthesis-related enzymes *PAD4* and *SID2* within the SA biosynthesis pathway [18, 71]. Additionally, upon detection of pathogen-associated molecular patterns in *Arabidopsis thaliana*, *CBP60g* and *SARD1* enhance SA production, with functional redundancy observed between the two genes [18]. *CBP60g* requires calmodulin binding to inhibit bacterial growth, whereas *SARD1* operates independently of calmodulin binding [18]. Furthermore, *CBP60g* is critical during the initial phase of the plant defense response, while *SARD1* is more important in the later stages [18]. The transcription factor TGA1/4 regulates SA synthesis by modulating *CBP60g* and *SARD1* expression [72]. SA promotes *NPR1* gene expression, which interacts with the TGA transcription factor (TGA 2/5/6) to control *PR* gene expression [12, 73]. *TGA2/5/6* genes exhibit functional redundancy and are essential for plant acquired resistance, exerting a negative regulatory effect on *PR* gene expression [13]. SA inhibits the expression of its receptors, *NPR3* and *NPR4*, while *NPR4* modulates the biosynthesis of SA [6, 11, 12]. *NPR3* and *NPR4* genes show functional redundancy and act antagonistically to *NPR1*, also interacting with TGA2/5/6 [12]. *MPK4* acts as a negative regulator of acquired resistance in plants and hampers the infection of *Arabidopsis thaliana* by *P. capsici* [14, 70]. The roles of *C3H14* in systemic acquired resistance (SAR) rely on phosphorylation mediated by *MPK4* [14, 70, 74]. Moreover, *MPK4* modulates SA- and JA-mediated defense responses by regulating *PAD4* and *EDS1* expression [15]. Mutations in the JA receptor *COI* result in insensitivity to JA and disruption of the JA signaling pathway [25, 26]. Furthermore, *JAR1* and *PDF1.2* in the JA pathway positively regulate the defense of *Arabidopsis thaliana* against *P. capsici* infestans [70]. The positions of these genes in the SA and JA signaling pathways and the relationships between each gene have previously been summarized by Zhang et al. [29]. Additionally, the *CaSBP12* gene is inducible by SA and methyl jasmonate [42]. Therefore, we evaluated the transcript levels of these genes under *CaSBP12* overexpression in *Arabidopsis*, *NaHG* overexpression in *Arabidopsis*, *CaSBP12* overexpression in the sid2-2, coi1-21, and coi1-22 backgrounds, and in hybrid lines overexpressing *NaHG* and *CaSBP12* in *Arabidopsis*. Then, based on our research results and the relationships between each gene as summarized by Zhang et al. [27], we speculated that the *CaSBP12* gene regulates plant defense by modulating defense-related gene expression (Fig. 9). Additionally, it downregulates the expression of the upstream genes *NDR1*, *PAD4*, and *EDS5* involved in SA biosynthesis, thereby reducing SA levels and consequently weakening the promotion of PR gene expression by SA, thus affecting plant defense responses (Fig. 9). Simultaneously, the *CaSBP12* gene upregulates the expression of *NPR1* while inhibiting the expression of *NPR3* and *NPR4* (Fig. 9). *NPR1* enhances *PR* gene transcription by interacting with TGA2/5/6, while *NPR3* and *NPR4* suppress *PR* gene expression by binding to *TGA2/5/6*. Therefore, suppressing *NPR3* and *NPR4* alleviates this inhibition, enhancing plant disease resistance. Accordingly, further research is needed to elucidate how the *CaSBP12* gene regulates plant defense responses. For instance, it should be determined whether the *CaSBP12* gene interacts with the four defense-related genes *CaPO1*, *CaBPR1*, *CaDEF1*, and *CaSAR8.2*, and if so, how it regulates these four defense-related genes in the plant defense response. Besides, it is necessary to supplement some data, such as the disease index situation of different strains shown in Fig. 5, to assist in explaining the differences in disease susceptibility among the strains. The effects of exogenous salicylic acid on the disease severity of various strains, clarifying the role of salicylic acid in the interaction process between pepper, *Arabidopsis*, and *Phytophthora capsici*. The current hypothesis suggests that *CaSBP12* inhibits the expression of *NDR1*, *PAD4*, and *EDS5*, thereby reducing SA production and subsequently inhibiting the expression of *PR* genes. Simultaneously, when SA is inhibited, it alleviates the repression of *NPR3/NPR4* on *PR* gene expression. However, *CaSBP12* also promotes SA production by inducing *SARD1* expression, thereby enhancing *PR* gene expression. Additionally, *CaSBP12* can promote *NPR1* expression, further enhancing *PR* gene expression. Therefore, the mechanism of this full regulation, both in promoting and inhibiting *PR* gene expressions, requires further investigation. Besides, our research can lay the foundation for further research on the molecular mechanisms by which *CaSBP12* participates in plant defense responses to *Phytophthora capsici* infection.

## Conclusions

Silencing the *CaSBP12* gene enhanced disease resistance in *CaSBP12*-silenced plants. Even in the absence of pathogen treatment, the defense-related genes *CaPO1*, *CaSAR8.2*, *CaBPR1*, and *CaDEF*1 showed significantly higher expression in *CaSBP12*-silenced plants compared to the controls. Conversely, these genes were downregulated in plants transiently expressing *CaSBP12*. Overexpression of *CaSBP12* in *Arabidopsis thaliana* increased the expression of the upstream SA signaling pathway genes *SARD1* and *SID2*, while downregulating the upstream genes *PAD4* and *NDR1* and the downstream genes *NPR3* and *NPR4*. In the sid2-2, coi1-21, and coi1-22 mutants overexpressing *CaSBP12*, genes associated with both SA and JA signaling pathways were differentially affected. These findings suggest that *CaSBP12* plays a crucial role in plant defense mechanisms, through its influence on the expression of defense-related genes and modulating genes within the SA signaling pathway. Further verification is needed to determine potential interactions between *CaSBP12* and SA signaling pathway genes to elucidate how *CaSBP12* participates in plant defense responses.

## Supplementary Information

Supplementary Material 1. Supplementary Table 1. Vectors' construct and quantitative PCR primer sets and their sequences. Supplementary Figure 1. Phenotype and silencing efficacy of CaSBP12-silenced plant. (A) Plant phenotype following* CaSBP12* silencing. Images obtained forty days post-injection, pot diameter 7 cm. (B) *CaSBP12* silencing efficacy in silenced versus negative control plants. ** denotes significant disparity at *P* < 0.01. Mean values and SDs for three replicates are displayed. Supplementary Figure 2. The expression levels of *AtETR1* and *AtTGA4* in *CaSBP12 *transgenic and wild type lines. ** denotes significance at *P* < 0.01. Mean values and SDs for three replicates are displayed. Supplementary Figure 3. Expression levels of salicylic acid signaling pathway-related genes in *NaHG* overexpressing strains, *NaHG *and *CaSBP12* co-expressing strains, sid2-2 strains and *CaSBP12* overexpression strains in sid2-2. Letters indicate significant differences at *P* < 0.05. Mean values and SDs for three replicates are displayed.

## Data Availability

All data pertinent to the current study have been provided in the manuscript or in the supplemental materials.
