# Supplementary material for: CaSBP12 is implicated in pepper’s defense resistance to Phytophthora capsici infection associated with the SA signaling pathway
Source: BMC Plant Biol. 2025 Dec 2;26:33. doi: 10.1186/s12870-025-07858-z (PMC12777433; doi:10.1186/s12870-025-07858-z)
Supplement: Supplementary file 1 — Supplementary Material 1. Supplementary Table 1. Vectors' construct and quantitative PCR primer sets and their sequences. Supplementary Figure 1. Phenotype and silencing efficacy of CaSBP12-silenced plant. (A) Plant phenotype following CaSBP12 silencing. Images obtained forty days post-injection, pot diameter 7 cm. (B) CaSBP12 silencing efficacy in silenced versus negative control plants. ** denotes significant disparity at P < 0.01. Mean values and SDs for three replicates are displayed. Supplementary Figure 2. The expression levels of AtETR1 and AtTGA4 in CaSBP12 transgenic and wild type lines. ** denotes significance at P < 0.01. Mean values and SDs for three replicates are displayed. Supplementary Figure 3. Expression levels of salicylic acid signaling pathway-related genes in NaHG overexpressing strains, NaHG and CaSBP12 co-expressing strains, sid2-2 strains and CaSBP12 overexpression strains in sid2-2. Letters indicate significant differences at P < 0.05. Mean values and SDs for three replicates are displayed. [file 12870_2025_7858_MOESM1_ESM.doc]

**SUPPLEMENTARY TABLES AND FIGURES**

**Supplementary Table 1.** Vectors' construct and quantitative PCR primer sets and their sequences.

| **Oligo Name** | **Primer Abbreviation** | **Primer Sequence (5^’-^3’)** |
| --- | --- | --- |
| ***CaSBP12*** | CaSBP12-2307-GFP-F | GCTCTAGAATGTTGGACTATGACTGGGGAG |
|  | CaSBP12-2307-GFP-R | GGGGTACCTGGTCTTTGCCTAAAACAATCC |
|  | CaSBP12-VIGS-F | CGGGATCCATCCTCCGTTATGCTTTCTGGC |
|  | CaSBP12-VIGS-R | GGGGTACCTACCTTGGGAATGGGTGAAACA |
|  | RTCaSBP12-VIGS-F | GTTTCACCCATTCCCAAGGTAATT |
|  | RTCaSBP12-VIGS-R | TAGTACGTCGGTAAAGTCGATTAACAA |
| ***NAHG*** | NAHG-F | GCTCTAGAATGAAAAACAATAAACTTGGCTT |
|  | NAHG-R | CGGGATCCTCACCCTTGACGTAGCGC |
| ***CaActin2*** | CaActin2-F | TCCACCTCTTCACTCTCTGCTC |
|  | CaActin2-R | TGACCCATCCCTACCATAACAC |
| ***CaPO1*** | CaPO1-F | GGCGCCAGGATTGCTGACAA |
|  | CaPO1-R | GTGGACATAATCCTCGAAGC |
| ***CaDEF1*** | CaDEF-F | CAAGGGAGTATGTGCTAGTGAGAC |
|  | CaDEF-R | TGCACAGCACTATCATTGCATAC |
| ***CaSAR8.2*** | CaSAR8.2-F | CAGGGAGATGAATTCTGAGGC |
|  | CaSAR8.2-R | CATATGAACCTCTATGGATTTCTG |
| ***CaBPR1*** | CaBPR1-F | CAGGATGCAACACTCTGGTGG |
|  | CaBPR1-R | ATCAAAGGCCGGTTGGTC |
| ***AtNPR1*** | AtNPR1-F | CGTCGCTACCGATAACAC |
|  | AtNPR1-R | AACCGACTTCGTAATCCTT |
| ***AtNPR3*** | AtNPR3-F | TTCACGGGTTTGTCACCTCC |
|  | AtNPR3-R | TCGACCAGTCTCAACTGTTTTC |
| ***AtNPR4*** | AtNPR4-F | TTCCCAGCAGAAGCCAATGT |
|  | AtNPR4-R | TTCCCAGCAGAAGCCAATGT |
| ***AtPR1*** | AtPR1-F | ACGGGGAAAACTTAGCCTGG |
|  | AtPR1-R | TTGGCACATCCGAGTCTCAC |
| ***AtPAD4*** | AtPAD4-F | TATGGTCGACGCTGCCATAC |
|  | AtPAD4-R | CACGTGGCAGAAGTTGTGTG |
| ***AtEDS1*** | AtEDS1-F | GAAGAAGCAGGAGCAGTCGT |
|  | AtEDS1-R | CCACAGAAGCTTGAAATGAGGT |
| ***AtMPK4*** | AtMPK4-F | AAGCTCGGGTGATCAAAGCA |
|  | AtMPK4-R | ATTTGAGCCCACGCAACAAC |
| ***AtEDS5*** | AtEDS5-F | TTCGGTCCTTGGGCTGTTAC |
|  | AtEDS5-R | CTGTGAAGCAGTTGTTGCCC |
| ***AtETR1*** | AtETR1-F | CCGGGGTCGAAAACTACCAA |
|  | AtETR1-R | GGTTTGAGCAACACACCGTC |
| ***AtPDF1.2*** | AtPDF1.2-F | TCTCTTTGCTGCTTTCGACG |
|  | AtPDF1.2-R | CCCTGACCATGTCCCACTTG |
| ***AtNDR1*** | NDR1-F | TGAAGACACAGAAGGTGGTCG |
|  | NDR1-R | GTCTTTTCCGAGGGCAGGAA |
| ***AtSARD1*** | SARD1-F | TCCTCTCGCCACATCAACAC |
|  | SARD1-R | GGCTCGCAGCATATTGTTGG |
| ***AtCBP60G*** | CBP60G-F | TGCCATGGATTGCGTTTTGG |
|  | CBP60G-R | GGATCCAAACTTCCTTGAAAGTCG |
| ***AtTGA2*** | AtTGA2-F | AATGCGCATGCAGGTGATTC |
|  | AtTGA2-R | TTGAACCAAGAGTCCCGCTC |
| ***AtTGA5*** | AtTGA5-F | AGGGCATTTGGGTATCGGTG |
|  | AtTGA5-R | GCTTTTCCTTGCAGCCTCAC |
| ***AtTGA6*** | AtTGA6-F | GAGCAAGACAGCAGGGAGTT |
|  | AtTGA6-R | ATGGGCTCTAGCTGATTCGC |
| ***AtTGA4*** | AtTGA4-F | TCGGATCTTAACCACGCGAC |
|  | AtTGA4-R | TACGTTGGTTCACGTTGCCT |
| ***AtActin8*** | AtActin8-F | CAACTATGTTCTCAGGTATTGCAGA |
|  | AtActin8-R | GTCATGGAAACGATGTCTCTTTAGT |

**
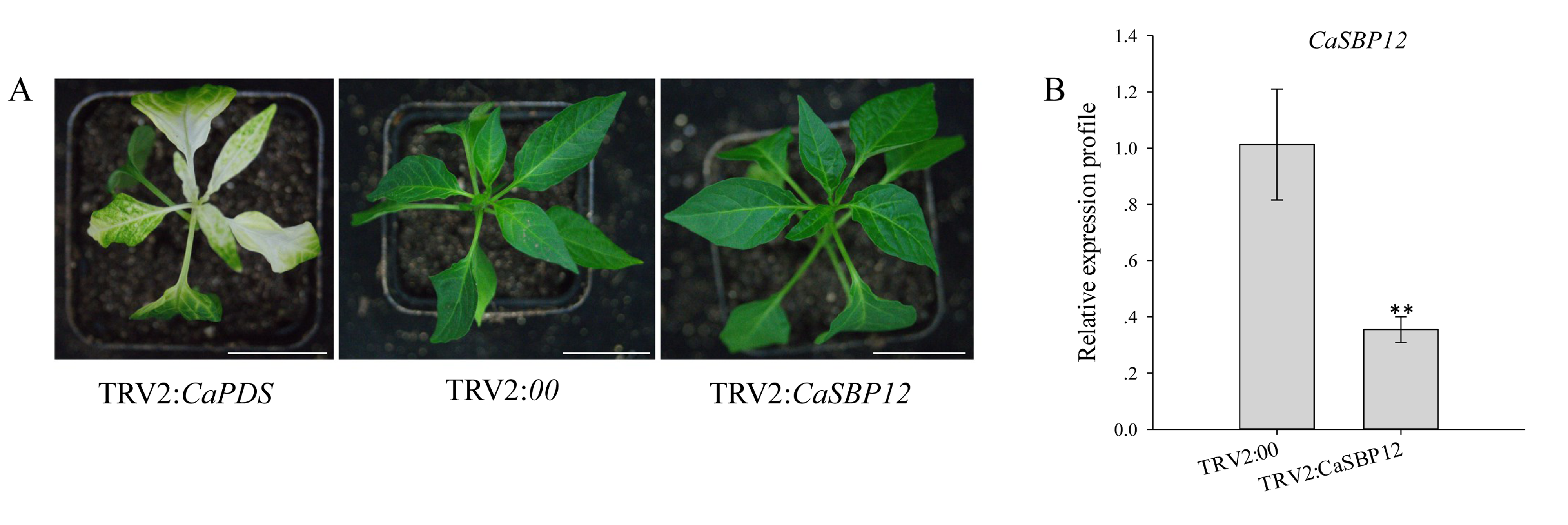
**

**Supplementary Figure 1.** Phenotype and silencing efficacy of CaSBP12-silenced plant. (A) Plant phenotype following *CaSBP12* silencing. Images obtained forty days post-injection, pot diameter 7 cm. (B) *CaSBP12* silencing efficacy in silenced versus negative control plants. ** denotes significant disparity at *P* < 0.01. Mean values and SDs for three replicates are displayed.

**
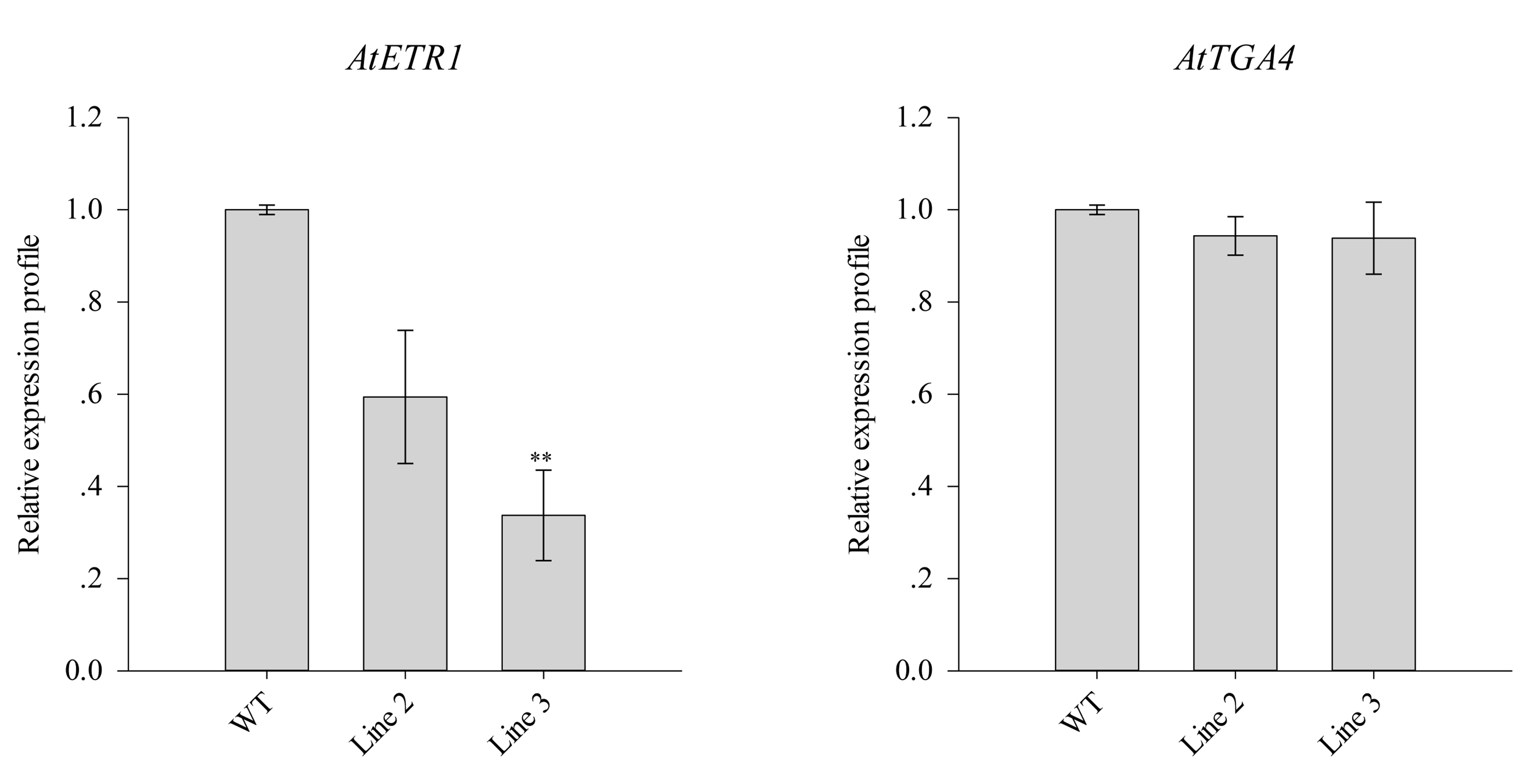
**

**Supplementary Figure 2.** The expression levels of *AtETR1* and *AtTGA4* in *CaSBP12* transgenic and wild type lines. ** denotes significance at *P* < 0.01. Mean values and SDs for three replicates are displayed.

**
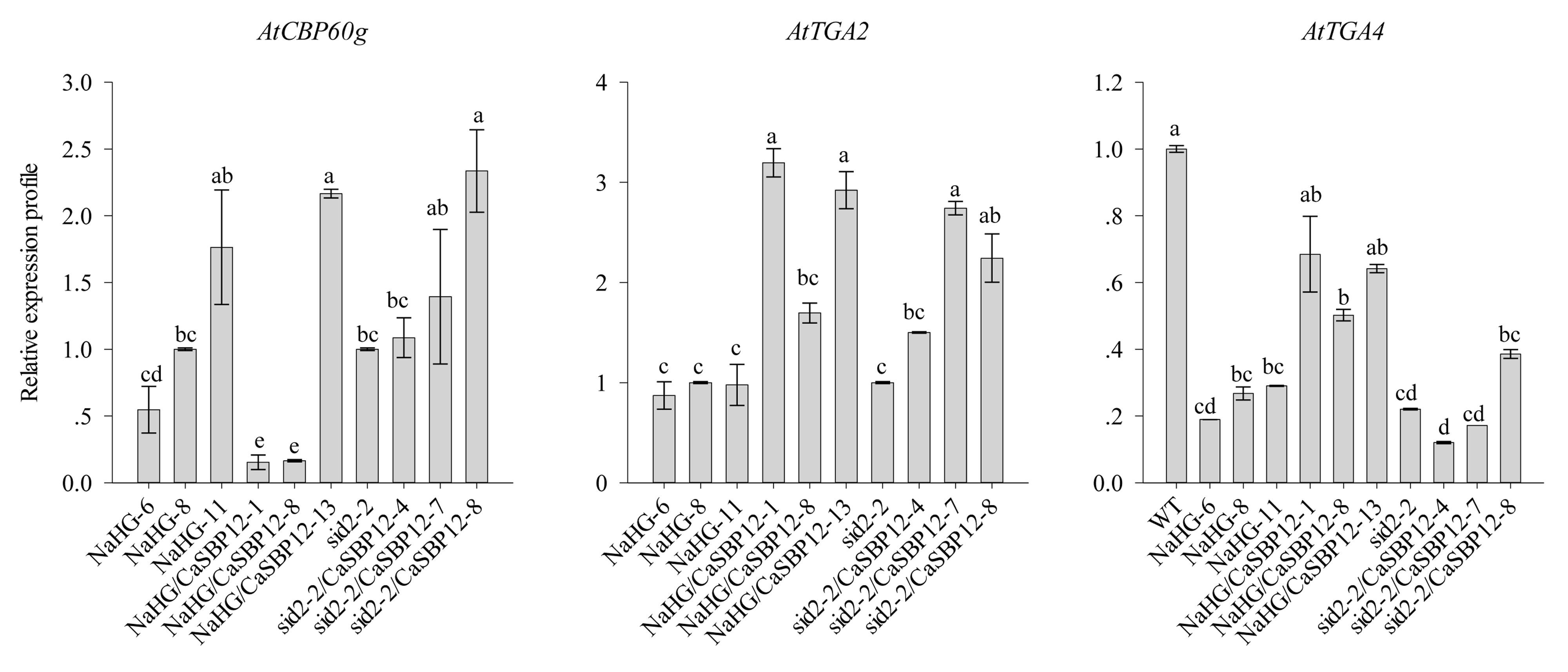
**

**Supplementary Figure 3.** Expression levels of salicylic acid signaling pathway-related genes in *NaHG* overexpressing strains, *NaHG* and *CaSBP12* co-expressing strains, sid2-2 strains and *CaSBP12* overexpression strains in sid2-2. Letters indicate significant differences at *P* < 0.05. Mean values and SDs for three replicates are displayed.
